# Pediatric sitosterolemia presenting with arthralgia and elevated inflammatory markers mimicking rheumatic disease: two case reports and a review of the literature

**DOI:** 10.3389/fped.2026.1966168

**Published:** 2026-10-28

**Authors:** Linxiaoyu Kong, Yihuai Xu, Xingcui Wang, Ruijuan Li

**Affiliations:** Department of Rheumatology and Immunology, Children’s Hospital Affiliated to Shandong University (Jinan Children’s Hospital), Jinan, Shandong, China

**Keywords:** arthralgia, case report, child, sitosterolemia, xanthoma

## Abstract

**Background:**

Sitosterolemia (STSL) is a rare autosomal recessive lipid-storage disorder caused by biallelic loss-of-function variants in ABCG5 or ABCG8, leading to intestinal hyperabsorption and reduced biliary excretion of plant sterols. It is phenotypically heterogeneous; xanthomas, haematological abnormalities and premature atherosclerosis are typical, whereas arthralgia is an under-recognised presenting feature that can mimic rheumatic disease.

**Case presentation:**

We report two boys (aged 14 and 8 years) who were first evaluated in a paediatric rheumatology department for arthralgia accompanied by cutaneous xanthomas and markedly elevated acute-phase reactants (C-reactive protein 45.8–95 mg/L; erythrocyte sedimentation rate 70–120 mm/h), initially raising suspicion of juvenile idiopathic arthritis. Both had marked hypercholesterolaemia and elevated low-density lipoprotein cholesterol, and serum sitosterol was strikingly raised (44.86 and 727.60 mg/dL; reference ≤0.501 mg/dL). Case 1 additionally showed mild anaemia, giant platelets on the blood smear, right coronary artery dilatation and increased carotid intima–media thickness. Whole-exome sequencing identified biallelic ABCG5 variants in both patients: a homozygous c.1336C > T (p.Arg446Ter) in Case 1, and compound heterozygous c.727C > T (p.Arg243Ter) and c.904 + 1G > A in Case 2; c.727C > T had not previously been reported in the Chinese population. After a low-phytosterol diet and ezetimibe, arthralgia resolved, xanthomas regressed, inflammatory markers normalised and platelet morphology improved over 6 months.

**Conclusions:**

Sitosterolemia can present with joint symptoms and a pronounced acute-phase response that mimics rheumatic disease. In children with unexplained arthritis accompanied by xanthomas, dyslipidaemia or cytopenias, serum phytosterol profiling and ABCG5/ABCG8 sequencing should be pursued.In these two patients, dietary phytosterol restriction combined with ezetimibe was associated with resolution of arthralgia, regression of xanthomas and normalisation of inflammatory markers over 6 months.

## Background

Sitosterolemia (STSL), first described by Bhattacharyya and Connor in 1974, is a rare autosomal recessive disorder characterised by elevated plasma phytosterol concentrations, xanthomatosis and premature atherosclerosis (1, 2). The disorder is caused by biallelic (homozygous or compound heterozygous) loss-of-function variants in ABCG5 or ABCG8, which encode the ATP-binding cassette (ABC) sterol transporters responsible for hepatic and intestinal sterol excretion. The resulting transporter dysfunction increases intestinal absorption and decreases biliary excretion of sterols, leading to abnormal accumulation and markedly elevated plasma concentrations of phytosterols, including sitosterol, campesterol and stigmasterol (3, 4). The estimated frequency of the causative variants in the general population is approximately 1 in 200,000 (5), and ABCG5 variants are more common among Chinese patients (6).

STSL is highly heterogeneous, ranging from asymptomatic disease to marked sterol accumulation in plasma and tissues causing xanthomatosis, hypercholesterolaemia, coronary atherosclerosis and sudden cardiac death in childhood; other manifestations include haemolytic anaemia, thrombocytopenia, arthralgia, arthritis, splenomegaly, hepatomegaly and hepatic failure (7–9). Because arthralgia may be the presenting complaint, such patients often attend a rheumatology department first and may be misdiagnosed as having juvenile idiopathic arthritis (JIA); the overlap with familial hypercholesterolaemia (FH) further complicates the diagnosis. As delayed diagnosis can lead to premature atherosclerotic cardiovascular disease, whereas early dietary management and pharmacotherapy (e.g., ezetimibe) substantially improve outcomes, increased clinical awareness of the diverse presentations of STSL is essential.

We retrospectively report two children with arthralgia-predominant STSL who were first evaluated in the Department of Rheumatology and Immunology of our hospital between 2024 and 2025, together with a review of the literature, in order to improve recognition of this atypical presentation and to reduce rates of missed and incorrect diagnosis.

## Case presentation

### Case 1

Case 1 was a 14-year-old boy admitted for joint swelling and pain for 5 days and fever for 1 day. He was the offspring of a non-consanguineous marriage, with no family history of cardiac disease and no history of trauma. On examination he was alert with unlaboured breathing. Firm, non-tender, pale-yellow papules and nodules were palpable over the left ear, both elbows and both knees. There was no lymphadenopathy. The lungs were clear. The heart rate was 78 beats/min with a regular rhythm, no pericardial friction rub and no murmur. The abdomen was soft, with no hepatosplenomegaly, and there was no skeletal deformity. The right knee was swollen, warm and tender with restricted movement; the other joints were normal.

### Case 2

Case 2 was an 8-year-old boy admitted for intermittent pain of both knees, both ankles and the right hip for 1 year. He was also born to non-consanguineous parents and had no history of trauma; his paternal grandmother had hyperlipidaemia. On examination he was alert with unlaboured breathing. A firm, non-tender nodule approximately 0.5 cm in diameter was present at the right ocular canthus, and hyperpigmentation without a palpable nodule was noted over the extensor surface of the left knee. There was no lymphadenopathy. The lungs were clear, and cardiac examination was unremarkable (heart rate 82 beats/min, regular, no murmur). The abdomen was soft without hepatosplenomegaly, and there was no skeletal deformity. Both ankles and knees were erythematous, swollen, warm and restricted in movement; the FABER (Patrick) test was positive on the right and negative on the left, and the patellar ballottement test was negative (Figure 1).

**Figure 1:**
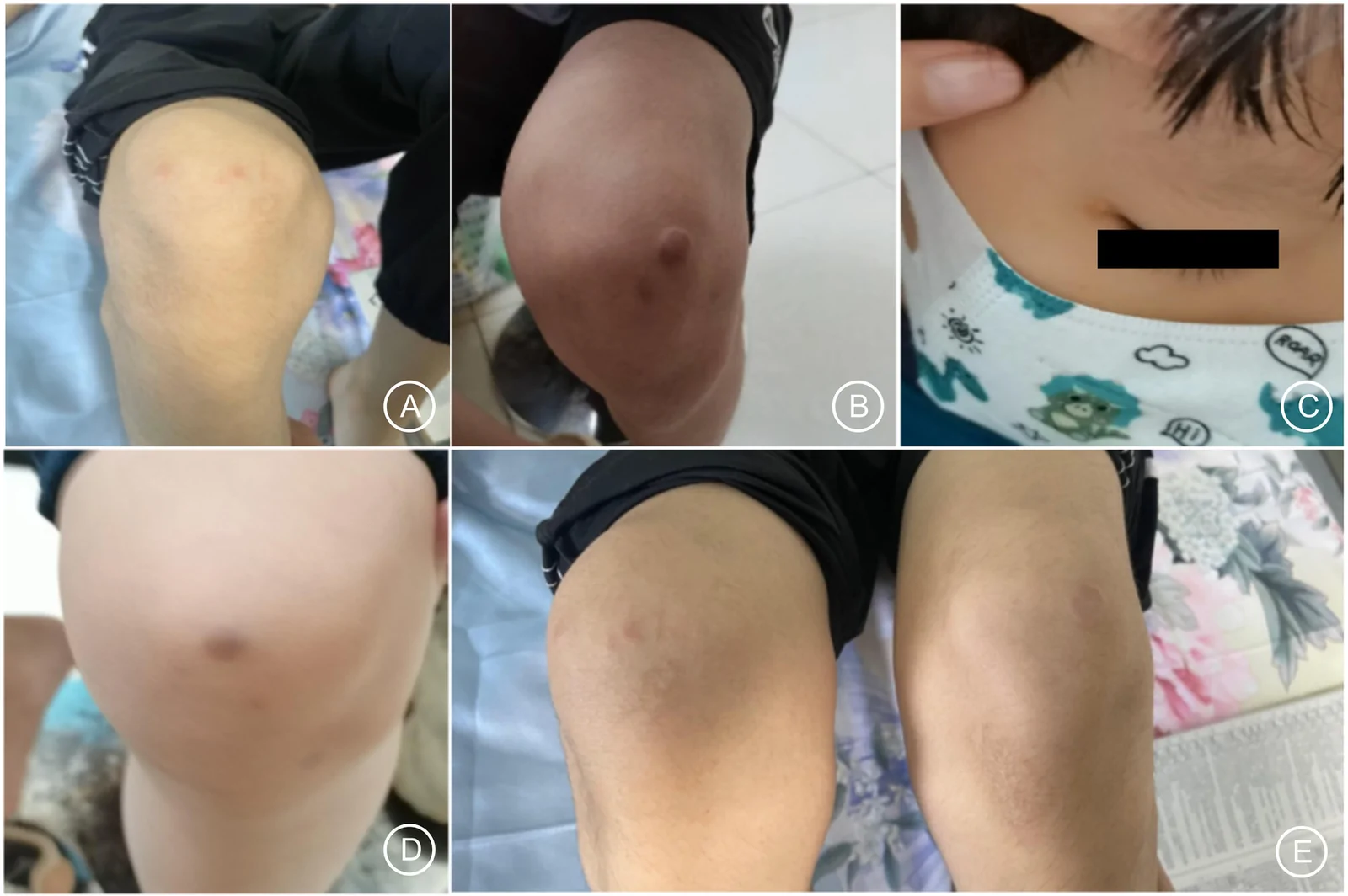
Cutaneous xanthomas in two children with sitosterolemia. **(A,B)** Xanthomas in Case 1 at initial presentation. **(C,D)** Xanthomas in Case 2 at initial presentation. **(E)** Marked regression of the xanthomas after 6 months of treatment.

In both patients, synovial fluid analysis, Gram stain, culture, and serological testing for common arthritogenic pathogens were not performed. Empirical antibiotic therapy (penicillin or second-generation cephalosporin) was administered for 3–5 days without clinical or laboratory improvement, and blood cultures were negative. Neither patient reported a history of trauma or preceding gastrointestinal or upper respiratory infection.

### Investigations

#### Haematology and biochemistry

In Case 1, the haemoglobin concentration was 102 g/L (mild anaemia) with otherwise normal values, and the peripheral blood smear showed large platelets with occasional giant platelets; the blood count was normal in Case 2. Both children had marked hypercholesterolaemia with elevated low-density lipoprotein cholesterol (LDL-C) (Table 1).

**Table 1:** Laboratory findings at presentation of the two patients at presentation.

Parameter	Reference range	Case 1	Case 2
Total cholesterol (mmol/L)	0–5.17	7.64	10.64
LDL-C (mmol/L)	0-3.36	5.89	8.33
CRP (mg/L)	0-10	95	45.8
ESR (mm/h)	0-15	120	70

LDL-C, low-density lipoprotein cholesterol; ESR, erythrocyte sedimentation rate.

#### Inflammatory and immunological markers

Acute-phase reactants were markedly elevated in both patients (Table 1). On repeated previous visits for arthralgia, Case 2 had shown C-reactive protein (CRP) fluctuating between 22.05 and 61.88 mg/L and an erythrocyte sedimentation rate (ESR) between 53 and 68 mm/h. Rheumatoid factor, complement, antinuclear antibody and anti-cyclic citrullinated peptide antibody were unremarkable in both children.

#### Cardiac and vascular ultrasound

Echocardiography demonstrated right coronary artery dilatation and aortic regurgitation in Case 1 and was normal in Case 2. Carotid ultrasound showed bilateral thickening of the common carotid arterial wall in both children, with additional brachiocephalic trunk wall thickening in Case 1; ultrasound of the limb vessels and abdominal great vessels was normal.

#### Joint imaging

In Case 1, magnetic resonance imaging (MRI) of the right knee showed injury of the quadriceps tendon and patellar ligament, injury of the medial patellar retinaculum, grade I injury of the posterior horn of the medial meniscus, a small joint effusion, and oedema of the infrapatellar fat pad and prepatellar subcutaneous soft tissue; knee ultrasound revealed symmetrical fusiform thickening of both quadriceps tendons, symmetrical thickening of the tibial insertions of both patellar ligaments, thickening of the left iliotibial tract, a small right knee effusion, and irregular margins of the medial and left lateral menisci. In Case 2, contrast-enhanced MRI of the hips, knees and sacroiliac joints showed abnormal signal within the epiphyses of the distal right femur and proximal tibia, linear synovial enhancement of the hip and knee joints, and slight blurring of the sacroiliac joints.

#### Other investigations

Chest computed tomography and brain MRI showed no space-occupying lesions in either child, and a bone-marrow smear in Case 1 revealed no abnormal cells. Ophthalmological examination demonstrated bilateral corneal opacity in Case 1.

#### Genetic testing

Whole-exome sequencing of the patients and their parents, with Sanger confirmation and family co-segregation analysis, identified biallelic pathogenic ABCG5 variants in both children (Table 2, Figure 2). Case 1 carried a homozygous c.1336C > T (p.Arg446Ter) variant inherited from both parents. Case 2 carried compound heterozygous variants: c.727C > T (p.Arg243Ter) inherited from the father and the c.904 + 1G > A splice-site variant inherited from the mother. The c.727C > T variant had not previously been reported in the Chinese population.

**Table 2:** ABCG5 variants identified in the two patients.

Patient	Variant 1 (nucleotide/protein)	Origin	Variant 2 (nucleotide/protein)	Origin
Case 1	c.1336C > T (p.Arg446Ter)	Paternal	c.1336C > T (p.Arg446Ter)	Maternal
Case 2	c.727C > T (p.Arg243Ter)	Paternal	c.904 + 1G > A (splice-site)	Maternal

Reference transcript NM_022436.3. The c.727C > T variant in Case 2 had not previously been reported in the Chinese population.

**Figure 2:**
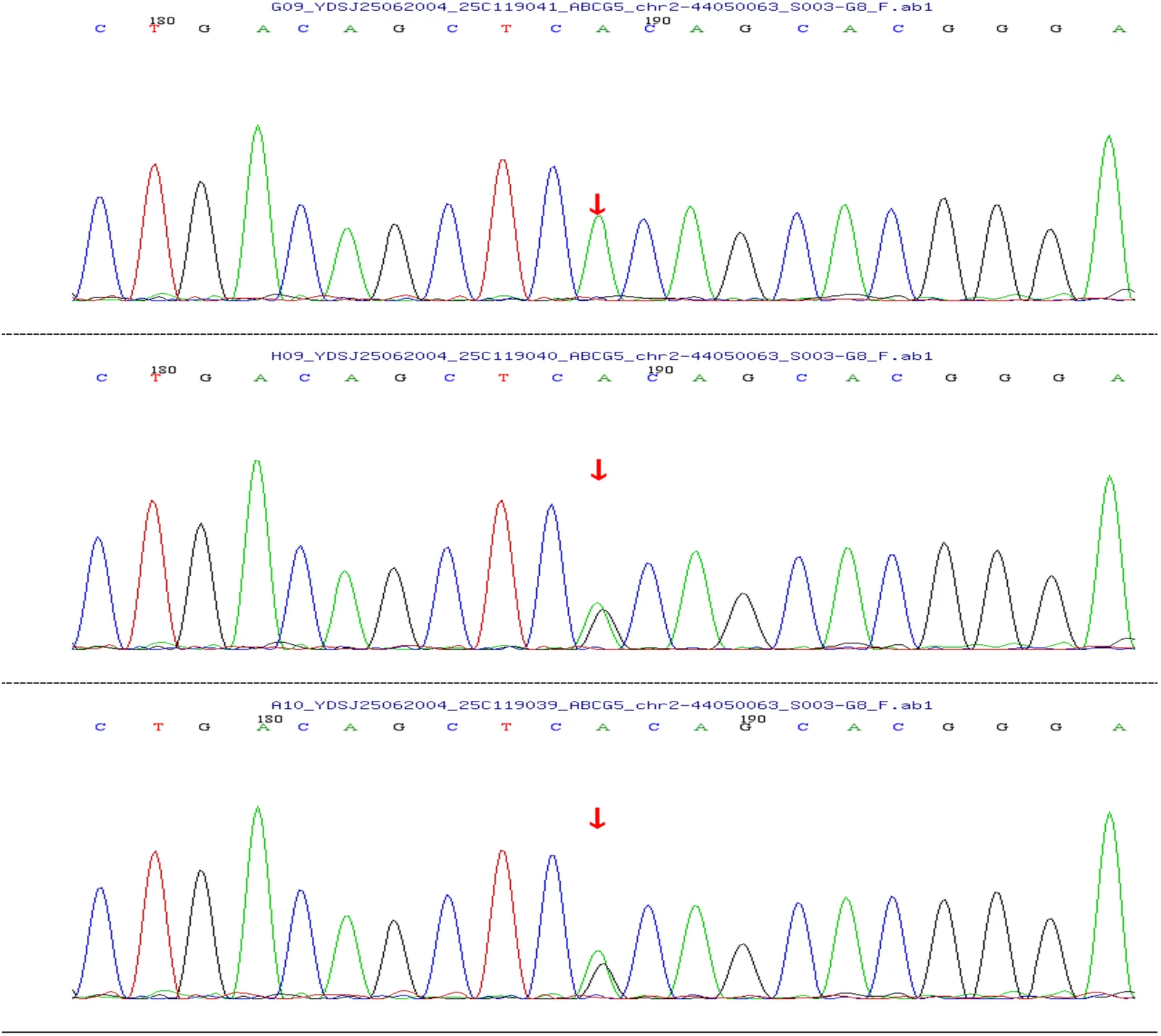
Sanger sequencing electropherograms of the ABCG5 c.1336C > T variant in case 1. **(A)** The proband carries the homozygous c.1336C > T variant in exon 10 of transcript NM_022436.3. **(B)** The father is a heterozygous carrier of c.1336C > T. **(C)** The mother is a heterozygous carrier of c.1336C > T.

#### Serum phytosterol profile

Gas chromatography–mass spectrometry (GC-MS) showed markedly elevated serum sitosterol and campesterol in both children, well above the upper reference limits (see Additional File S1: Supplementary Table S1 for the complete sterol profile). Variant classification followed ACMG/AMP guidelines; detailed classification evidence for each variant is provided in Additional File S2: Supplementary Table S2.

### Treatment and follow-up

The key clinical events for each patient, from symptom onset through diagnosis, treatment initiation and follow-up, are summarised in Additional File 3: Supplementary Table S3. After diagnosis, both children received dietary phytosterol restriction and oral ezetimibe 10 mg once daily (Case 1: 14 years; Case 2: 8 years); a nonsteroidal anti-inflammatory drug was used briefly for acute arthralgia and discontinued within 1 month. Over 6 months of follow-up, the cutaneous xanthomas regressed markedly (Figure 1) and arthralgia resolved completely in both patients, with normalisation of CRP. In Case 1, the anaemia resolved and the giant platelets disappeared from the peripheral smear. At the 6-month follow-up, serum phytosterol and lipid levels were not routinely re-measured, as the primary clinical response (resolution of arthralgia, regression of xanthomas, normalisation of CRP) was evident. However, given the importance of biochemical monitoring in STSL, future follow-up will include periodic assessment of phytosterol and lipid profiles.

## Discussion

STSL results from homozygous or compound heterozygous variants in ABCG5 or ABCG8, leading to increased intestinal absorption and reduced biliary excretion of phytosterols. In healthy individuals, dietary phytosterols have no physiological role, and their plasma concentration is normally less than 0.5% of that of cholesterol (10); by contrast, patients with STSL absorb 15%–60% of ingested phytosterols, increasing plasma phytosterol concentrations 50- to 200-fold (2). A plasma sitosterol concentration above 1 mg/dL (10 µg/mL) is considered diagnostically significant, consistent with the values in our two patients.

The two cases presented here, despite sharing the same genetic defect, exhibited striking phenotypic discordance: Case 1 (homozygous p.Arg446Ter) had relatively lower plasma sitosterol (44.86 mg/dL) but developed coronary artery dilatation and haematological abnormalities, while Case 2 (compound heterozygous) had a 16-fold higher sitosterol level (727.60 mg/dL) yet no cardiovascular or haematological involvement at presentation. This disparity, together with the prominent joint symptoms and acute-phase inflammatory response seen in both patients, raises three clinically relevant questions: (1) what mechanisms link phytosterol accumulation to systemic inflammation and arthritis that so closely mimic rheumatic disease? (2) why does the plasma phytosterol level not correlate with cardiovascular severity? and (3) how can clinicians avoid misdiagnosing STSL as juvenile idiopathic arthritis or enthesitis-related arthritis? We address these questions below in the context of our findings and the existing literature.

Joint symptoms have been reported in 14% to 27% of paediatric patients with STSL at diagnosis, indicating that articular involvement is not a rare feature of this metabolic disorder (11, 12). However, the pronounced acute-phase inflammatory response observed in our two cases has rarely been emphasised in previous reports. The mechanisms linking phytosterol accumulation to joint symptoms and systemic inflammation warrant consideration. While cholesterol crystals have been definitively shown to activate the NLRP3 inflammasome in macrophages, promoting the release of pro-inflammatory cytokines IL-1β and IL-18 (13), direct evidence that phytosterols activate the same pathway is currently lacking. We therefore hypothesise that, by analogy with cholesterol crystals, phytosterol accumulation in STSL may create crystal- or membrane-associated DAMPs capable of priming the NLRP3 inflammasome. If this proposed mechanism is operative, the downstream release of IL-1β could provide a mechanistic link between phytosterolaemia and the synovial inflammation, fever and hepatic CRP synthesis observed in our patients, independent of an autoimmune mechanism. This speculative cascade might also help explain the joint symptoms observed in our patients. It is also consistent with the clinical observation that the joint symptoms improved following lipid-lowering therapy alone, a pattern not typical of immune-mediated arthropathies such as enthesitis-related arthritis. However, confirmation of this pathway in STSL will require dedicated experimental studies.

### Attribution of joint symptoms to STSL

The joint manifestations in our patients, although atypical in pattern, occurred in close temporal association with the diagnosis of STSL and improved following lipid-lowering therapy. However, STSL classically causes chronic arthralgia or periarticular xanthoma deposition rather than acute inflammatory arthritis (11, 12), and the acute febrile monoarthritis in Case 1 and the synovial enhancement in Case 2 are more consistent with infectious, reactive or traumatic aetiologies. Although antibiotic therapy was ineffective, blood cultures were negative, and neither patient reported preceding trauma or infection, these findings cannot definitively exclude alternative causes given the absence of synovial fluid analysis. We therefore describe the joint symptoms as temporally associated with STSL rather than causally attributed to it.

Additionally, both patients had evidence of phytosterol-induced haematological abnormalities. Plant sterol accumulation in erythrocyte membranes increases their rigidity and osmotic fragility (14, 15), contributing to haemolysis and morphological changes. Accordingly, thrombocytopenia and haemolytic anaemia are common phenotypes of STSL (9, 11, 16). Case 1 had mild anaemia, and although thrombocytopenia was absent, giant platelets on the peripheral smear indicated that the elevated phytosterols had already affected platelet morphology.

Cutaneous xanthomas are the most common and frequently the first manifestation in paediatric STSL, with a reported frequency of 73.1%–100% (4); both of our patients presented with xanthomas at diagnosis, consistent with previous Chinese paediatric series (7, 12). Importantly, the regression of xanthomas paralleled the resolution of arthralgia and normalisation of inflammatory markers following dietary restriction and ezetimibe, suggesting a common pathogenic link between tissue sterol deposition and systemic inflammation.

Our two cases further illustrate the marked phenotypic heterogeneity of STSL. Despite both carrying biallelic ABCG5 variants, Case 1 (homozygous p.Arg446Ter) developed coronary artery dilatation and haematological abnormalities with a relatively lower sitosterol level, whereas Case 2 (compound heterozygous) had a 16-fold higher sitosterol concentration but no cardiovascular or haematological involvement at presentation. Previous genotype–phenotype analyses have demonstrated significant inter-individual variability in platelet count, haemoglobin and sitosterol concentration, even among patients carrying identical mutations (17); some homozygotes remain nearly asymptomatic, while others develop life-threatening atherosclerosis in childhood (2, 10). While no conclusion regarding the relationship between plasma phytosterol levels and cardiovascular severity can be drawn from two cases, the discordance observed here is consistent with the imperfect genotype–phenotype correlation in STSL. Factors beyond the primary genetic defect — including the duration of sterol exposure, residual transporter activity determined by specific mutation types (18), dietary phytosterol intake (19, 20), and potential modifier genes — may contribute to the clinical phenotype. Both patients carried ABCG5 variants, consistent with reports in Chinese patients (8, 21). The c.727C > T (p.Arg243Ter) variant identified in Case 2 has been reported in Indian and South African Asian populations (22), but to our knowledge, this is the first report in a Chinese patient, expanding the mutation spectrum of STSL in Chinese patients.

The marked phenotypic heterogeneity of STSL contributes to frequent diagnostic delays. The diagnosis of STSL is frequently delayed or overlooked, as its manifestations overlap with those of familial hypercholesterolaemia (FH). Both conditions present with hypercholesterolaemia and xanthomas; however, several features help distinguish STSL from FH. In contrast to FH, STSL is often accompanied by haematological abnormalities — including haemolytic anaemia, thrombocytopenia, and giant platelets — and shows a poor lipid-lowering response to statin therapy, whereas ezetimibe is highly effective (23, 24). A mean platelet volume ≥9.05 fL in a child with xanthomas has been proposed as a simple and valuable screening indicator for STSL (8). For paediatric rheumatologists, the following clinical clues should raise suspicion of STSL in a child presenting with arthralgia or joint symptoms: (1) cutaneous or tendon xanthomas, particularly when out of proportion to the LDL-C level; (2) unexplained haematological abnormalities, such as anaemia, thrombocytopenia or giant platelets on peripheral smear; (3) markedly elevated inflammatory markers (CRP/ESR) that do not respond to NSAIDs as expected; and (4) a family history of hyperlipidaemia or premature cardiovascular disease without a confirmed monogenic cause. When these features are present, serum phytosterol profiling and ABCG5/ABCG8 sequencing should be pursued promptly to confirm the diagnosis and avoid unnecessary immunosuppressive therapy.

The cornerstone of STSL management is dietary phytosterol restriction, which involves limiting non-cholesterol sterols, shellfish, high-fat plant foods and vegetable oils. As first-line pharmacotherapy, ezetimibe inhibits the Niemann–Pick C1-like 1 (NPC1L1) transporter and effectively reduces intestinal phytosterol absorption, with reported benefits including lowered plasma phytosterol concentrations, regression of xanthomas, and improvement in haemolytic anaemia and platelet abnormalities (11, 25, 26). Although ezetimibe is generally approved for children older than 10 years, accumulating evidence supports its safety and efficacy in younger children (11, 27); both of our patients (aged 14 and 8 years) received ezetimibe together with dietary restriction and showed marked clinical improvement without adverse effects over 6 months of follow-up. Bile-acid sequestrants such as cholestyramine represent a second-line option that can reduce plasma phytosterols by up to 45%, but their utility is limited by gastrointestinal side effects and poor adherence (28). Given that STSL is a treatable disorder, early diagnosis and prompt initiation of dietary and pharmacological intervention are essential to prevent premature atherosclerotic cardiovascular disease (5).

## Conclusions

Paediatric STSL can present with joint symptoms and a marked acute-phase inflammatory response, closely mimicking rheumatic disease such as JIA or ERA. Rheumatologists should maintain a high index of suspicion when xanthomas, unexplained haematological abnormalities, or dyslipidaemia accompany joint symptoms — particularly when inflammatory markers are disproportionately elevated or fail to respond as expected to NSAIDs. Serum phytosterol profiling and ABCG5/ABCG8 sequencing provide timely diagnostic confirmation and can prevent unnecessary immunosuppressive therapy. In our two patients, early dietary phytosterol restriction combined with ezetimibe was associated with resolution of arthralgia, regression of xanthomas and normalisation of inflammatory markers over 6 months. Although cardiovascular outcomes were not assessed in this short follow-up, early diagnosis and treatment may help reduce the long-term risk of premature atherosclerosis, as suggested by previous literature, underscoring the importance of prompt recognition and intervention in this rare but treatable disorder.

## Data Availability

The original contributions presented in the study are included in the article/Supplementary Material, further inquiries can be directed to the corresponding author.
