# Supplementary material for: Pediatric sitosterolemia presenting with arthralgia and elevated inflammatory markers mimicking rheumatic disease: two case reports and a review of the literature
Source: Front Pediatr. 2026 Oct 28;14:1966168. doi: 10.3389/fped.2026.1966168 (PMC13752081; doi:10.3389/fped.2026.1966168)
Supplement: Supplementary file 1 [file Table1.docx]

**Table S1** Serum sterol profile of the two patients measured by GC-MS (mg/dL)

|  | **Cholesterol** | **Cholestanol** | **Sitosterol** | **Campesterol** |
| --- | --- | --- | --- | --- |
| Reference range | ≤802.141 | ≤0.779 | ≤0.501 | ≤0.842 |
| Case 1 | 1050.56 | 15.41 | 44.86 | 21.93 |
| Case 2 | 1838.74 | 32.76 | 727.60 | 34.27 |

*GC-MS, gas chromatography–mass spectrometry.*
