# Supplementary material for: Pediatric sitosterolemia presenting with arthralgia and elevated inflammatory markers mimicking rheumatic disease: two case reports and a review of the literature
Source: Front Pediatr. 2026 Oct 28;14:1966168. doi: 10.3389/fped.2026.1966168 (PMC13752081; doi:10.3389/fped.2026.1966168)
Supplement: Supplementary file 2 [file Table2.docx]

**Table S2** ACMG/AMP classification of ABCG5 variants

| **Variant** | **Type** | **ACMG criteria** | **Classification** | **Supporting evidence** |
| --- | --- | --- | --- | --- |
| c.1336C>T (p.Arg446Ter) | Nonsense | PVS1+  PM3_VeryStrong+  PM2_Supporting | Pathogenic | PVS1: null variant (nonsense), predicted to cause loss of function;  PM3_VeryStrong: detected in multiple patients with xanthomatosis in trans with pathogenic/likely pathogenic variants (PMID: 19111681, 20719861, 24166850, 26813946, 28521186);  PM2_Supporting: population frequency 0.0008704 |
| c.727C>T (p.Arg243Ter) | Nonsense | PVS1+  PM2_Supporting+  PM3_Strong | Pathogenic | PVS1: null variant (nonsense), predicted to cause loss of function;  PM2_Supporting: population frequency 0.0001;  PM3_Strong: previously reported in patients with sitosterolemia (ClinVar: Pathogenic, Sitosterolemia 2; DM tag), detected in ≥4 homozygous sitosterolemia patients (PMID: 11138003, 29102041) |
| c.904+1G>A | Splice-site | PVS1+  PM2_Supporting+  PM3_VeryStrong | Pathogenic | PVS1: null variant (splice-site), predicted to cause loss of function;  PM2_Supporting: population frequency 0.0003262;  PM3_VeryStrong: previously reported in patients with sitosterolemia (DM tag; ClinVar no classification), recorded in MyGenostics case database (7 cases), detected in ≥4 hypercholesterolaemia patients in trans with pathogenic/likely pathogenic variants |

ACMG, American College of Medical Genetics and Genomics; AMP, Association for Molecular Pathology; DM, disease-causing mutation. Classification according to Richards et al.
