# Supplementary material for: Pediatric sitosterolemia presenting with arthralgia and elevated inflammatory markers mimicking rheumatic disease: two case reports and a review of the literature
Source: Front Pediatr. 2026 Oct 28;14:1966168. doi: 10.3389/fped.2026.1966168 (PMC13752081; doi:10.3389/fped.2026.1966168)
Supplement: Supplementary file 3 [file Table3.docx]

**Table S3** Timeline of key clinical events

|  | **Case 1** | **Case 2** |
| --- | --- | --- |
| Symptom onset | Joint swelling/pain for 5 days; fever for 1 day | Intermittent knee/ankle/hip pain for 1 year |
| Initial evaluation | Rheumatology outpatient | Rheumatology outpatient |
| Key findings at presentation | Xanthomas (ear, elbows, knees); right knee arthritis | Xanthoma at right ocular canthus; bilateral ankle/knee arthritis |
| Diagnostic evaluation | Lipid profile,  inflammatory markers,  echocardiography,  carotid ultrasound,  knee MRI,  genetic testing | Lipid profile,  inflammatory markers,  hip/knee/sacroiliac MRI,  genetic testing |
| Treatment initiation | Low-phytosterol diet +  ezetimibe 10 mg/day +  short-course NSAID | Low-phytosterol diet +  ezetimibe 10 mg/day +  short-course NSAID |
| 6-month follow-up | Arthralgia resolved;  xanthomas regressed;  CRP normalised;  anaemia and giant platelets resolved | Arthralgia resolved;  xanthomas regressed;  CRP normalised |
